# Psychological well‐being of fathers with and without a child with intellectual disability: a population‐based study.

**DOI:** 10.1111/jir.12692

**Published:** 2019-11-20

**Authors:** E. Langley, V. Totsika, R. P. Hastings

**Affiliations:** ^1^ Centre for Educational Development, Appraisal and Research (CEDAR) University of Warwick Coventry UK; ^2^ Division of Psychiatry, Faculty of Brain Sciences University College London London UK

**Keywords:** families, fathers, intellectual disability, population sample, well‐being

## Abstract

**Background:**

Few studies have explored the well‐being of fathers of children with intellectual disability (ID), despite the significant role that they play in their children's lives. The current study compared fathers of children with and without a child with ID on measures of psychological well‐being (life satisfaction, work–family balance and general health) and dimensions of parenting (parenting self‐efficacy and parent–child closeness) and then examined whether the presence of a child with ID in the family was a significant predictor of paternal well‐being when controlling for a number of father (age, education, employment and residency), child (ID status, gender, behavioural and emotional problems) and family (income poverty and number of children in the household) variables.

**Methods:**

Data were drawn from the third wave of the Millennium Cohort Study, a UK population‐representative and cohort study, where the cohort child was 5 years of age; 256 fathers were identified as having a child with ID, with data available for 10 187 fathers without a child with ID. Fathers were compared on the four well‐being and parenting outcomes and then multiple regression models were conducted to explore associations between these outcomes and variables identified as potential correlates of well‐being.

**Results:**

Initial group comparisons showed that there were differences in the well‐being of fathers, with fathers of children with ID reporting poorer life satisfaction and general health. However, these differences were small. Regression analyses showed that child behavioural and emotional problems, living in income poverty and paternal employment were more important than disability status in predicting fathers' well‐being.

**Conclusions:**

These works add to the limited amount of research on fathers using population‐representative data. The current findings are consistent with rejecting a general simplistic and negative narrative that raising a child with ID puts fathers at risk of poorer outcomes. However, some fathers, such as those with children with behavioural problems and living in poverty, may require greater support. Future longitudinal research that explores the impact of paternal well‐being on the long‐term outcomes of children with and without ID is warranted.

## Background

Theoretical frameworks such as family systems theory recognise that fathers are an integral part of the family unit (Seligman and Darling, 2007). The birth and care of a child with an intellectual disability (ID) is understood to affect every member of the family, including fathers, yet few studies have explored fathers' well‐being (Braunstein *et al.,* 2013; Macdonald and Hastings, 2010; Marquis *et al.,* 2019; Taylor *et al*. 2016). Parental well‐being is also considered a key determinant of child outcomes: the developmental systems model (Guralnick, 2001, 2005) describes how parental stress can be a risk factor for child development, indicating that paternal outcomes should be studied not only for their relevance to fathers but also to their children's outcomes. Research on mothers is driven by the premise that mothering influences children's outcomes (Pleck 2012), and this could be the same for fathers given their role in the family.

Fathers' well‐being has often been compared with that of mothers (Olsson and Hwang, 2001), with relatively few studies comparing psychological well‐being between fathers of children with and without ID. Like mothers of children with ID, it is important to compare fathers of children with ID with fathers of children without ID to better understand whether they report poorer well‐being outcomes and how we can best support them and their family. Group comparisons are needed to contextualise fathers' experiences (i.e. in relation to other fathers). Existing work suggests that fathers of children with developmental disabilities including ID are at heightened risk of experiencing mental health difficulties compared with fathers of children without a disability (Oelofsen and Richardson, 2006) and report significantly more symptoms of depression, anxiety and stress when compared with normative data (Giallo *et al.,* 2015). Seymour *et al*. (2017) compared the psychological well‐being of fathers of children with autism, other long‐term disabilities and without disabilities using Australian population‐representative data and found that while the majority of fathers experienced good psychological health, a considerable proportion of fathers of children with autism (17%) reported symptoms of psychological distress in the clinical ranges.

Research in this area has tended to explore more negatively focused, mental health problems such as depression and stress. However, it is important that an exploration of paternal well‐being also explores other dimensions. As defined by the World Health Organisation, mental health and well‐being is a ‘state of complete physical, mental and social wellbeing and not merely the absence of disease or infirmity' (World Health Organisation, 2014, para. 2). While mental health problems and subjective well‐being are correlated, there is evidence from research in the general population to suggest that there are distinct causal and mediating pathways for each of these concepts, which would require separate and targeted intervention (Kinderman *et al.,* 2015).

Subjective well‐being and, in particular, life satisfaction of fathers raising a child with ID has received little attention within the literature. However, fathers of children with ID may be more likely to face additional challenges that have an impact on their outlook on life. Some, albeit limited, evidence on life satisfaction suggests that fathers of children with ID do report lower levels of life satisfaction. Darling *et al*. (2012) compared the life satisfaction of 85 fathers of children with disabilities (the most common disability being attention deficit disorder, 36.5%) and 121 fathers of children without a disability using the 5‐item Satisfaction with Life Scale (Diener *et al.,* 1985). Results showed that fathers of children without a disability reported greater levels of life satisfaction than fathers of children with a disability.

The World Health Organisation definition of mental health and well‐being also includes an individual being able to ‘cope with the normal stresses of life' and ‘work productively and fruitfully' (WHO 2014 para. 1). Employment and work–family conflict have been found to predict the mental health outcomes of fathers of children without a disability (Cooklin *et al.,* 2015). Achieving a balance between work and family life may be more difficult when raising a child with ID due to additional caring responsibilities. There is evidence to suggest that raising a child with ID can have an impact upon work but also that work can impact upon a father's ability to care for their child (Davys *et al.,* 2017). Couples tend to become more differentiated in their work and family roles when they become parents, with men more likely to be the main breadwinner (Katz‐Wise *et al.,* 2010). Fathers may therefore experience difficulties in balancing their work and home lives.

Research with fathers of children with disabilities has found that inflexible work arrangements affect their ability to engage in the care of their child (Carpenter and Towers, 2008; Wright *et al.,* 2016), with fathers in lower paid and skilled jobs most affected (Carpenter and Towers, 2008). Job quality (flexibility and paid leave) has also been found to predict psychological distress in fathers of children with autism (Seymour *et al.,* 2018). It is important to further understand the impact that raising a child with ID has on the work–family balance of fathers and the factors predicting this outcome, as very little is currently known.

There is also a need to explore the physical health of fathers. Fathers of children with a disability may be more likely to put the needs of their family before their own, employing less self‐care and have been found to report poorer global health than fathers of children without disabilities (Seymour *et al.,* 2017). Poor general health has the potential to affect the daily functioning of fathers and their ability to care for their child with ID and/or any other children in the family.

The developmental systems model suggests that parenting and parent–child relationships mediate the impact of parental stress on child outcomes (Guralnick, 1997) and have been found to impact children's outcomes across a range of disabilities including ID (Taylor *et al.,* 2016; Totsika *et al.,* in press). Therefore, father–child relationships are important to explore in relation to the well‐being of fathers and their children. Raising a child with a disability may pose additional challenges that impact upon how competent an individual feels in their parenting role and the relationship they have with their child. Parenting self‐efficacy is defined as ‘an individual's appraisal of his or her competence in the parental role' (Sevigny and Loutzenhiser, 2010, p. 179). A child with ID is likely to have additional needs that require greater skills or expertise that influence how parents perceive their ability to meet these needs. Specific aspects of a child's phenotype, such as behavioural and emotional problems, are more likely to challenge families (Taylor *et al.,* 2016) and have been associated with poorer parenting self‐efficacy (Hassall *et al.,* 2005). These challenges also have the potential to affect the closeness of parent–child relationships, although it could conversely be argued that fathers of children with ID are more involved in the care of their child because of their additional needs that may then positively influence parenting self‐efficacy and the closeness of the relationship with their child. These aspects of parenting have received little attention in the literature (Dempsey *et al.,* 2009), particularly in regard to fathers. Further research is required to establish whether fathers of children with ID do report different levels of parenting self‐efficacy and father–child closeness compared with fathers without a child with ID, and what factors are associated with these outcomes to inform father–child interventions.

In addition to a narrow operationalisation of well‐being, much of the existing evidence on fathers of children with ID is based on small, convenience samples with findings that cannot generalise to the general population of fathers of children with ID. More recent studies have recognised the methodological and clinical limitations of small and unrepresentative samples and have used large population‐representative samples such as the Longitudinal Study of Australian Children to research fathers (Seymour *et al.,* 2017). In the United Kingdom, studies using data from the Millennium Cohort Study (MCS) have compared psychological distress levels in mothers and fathers with and without children with early cognitive delay at age 3 and age 5 (Emerson *et al.,* 2010). However, to date, the MCS has not been used to compare fathers of children with and without ID on other father well‐being and parenting dimensions.

An additional question is what factors may be associated with well‐being in fathers. Children with ID have been found to exhibit higher levels of behavioural and emotional problems compared with children with ID (Emerson and Hatton, 2007) and have poorer behavioural trajectories over time (Bailey *et al.,* in press). Increased levels of child behavioural and emotional problems have typically been associated with lower levels of well‐being in fathers of children with pervasive developmental disorder/autism (Herring *et al.,* 2006; Davis and Carter, 2008; Brobst *et al.,* 2009), ID (Giallo *et al.,* 2015; Cohen *et al.,* 2016) and Down syndrome (Ricci and Hodapp, 2003).

Other more distal environmental factors may also put fathers at risk of poorer outcomes, with one of the most significant being income poverty (Marquis *et al.,* 2019). The developmental systems model postulates that living in poverty and low levels of parental education are factors associated with ‘non‐optimal' levels of child development (Guralnick, 1997). Families of children with ID are at increased risk of economic deprivation compared with families of children without ID (Emerson, 2003). Evidence so far has established an association between socio‐economic deprivation and well‐being in mothers of young children at risk of a disability (Emerson and Llewellyn, 2008). A study in 2010 using MCS data at wave 2 (age 3) and wave 3 (age 5) also showed that socio‐economic deprivation accounted for differences in psychological distress between parents of children with and without early cognitive delay (Emerson *et al.,* 2010). Giallo *et al*. (2015) did not find an association between socio‐economic status and symptoms of depression, anxiety and stress in fathers of children with ID. However, this could be attributed to the use of a measure of area level of deprivation as opposed to individual level deprivation measures used in previous studies. More recent findings have also suggested that grouping individual‐level deprivation indicators in a composite may mask the different associations between well‐being and specific socio‐economic indicators, such as poverty or unemployment (Totsika *et al.,* 2016). Therefore, in the current analysis, we explored the association of paternal well‐being with specific indicators of socio‐economic position (SEP).

The aim of the present study was to explore the psychological well‐being of fathers with and without a child with ID, drawing upon UK population‐representative data. We focused on the following research questions:

- How do fathers of children with and without ID compare on measures of parental well‐being (life satisfaction, work–family balance and general health) and dimensions of parenting (parenting self‐efficacy and father–child closeness)?
- Is the presence of a child with ID in the family a significant predictor of paternal well‐being when controlling for a number of variables identified as potential correlates of well‐being?

## Methods

This study used data from wave 3 of the MCS when the cohort child was 5 years old (Centre for Longitudinal Studies, 2017). MCS is a longitudinal birth cohort study tracking the lives of approximately 19 000 British children who were born in the United Kingdom in 2000–2001 (see www.cls.ioe.ac.uk). Families were randomly selected using the Child Benefit register, which at the time of the study design was a non‐means‐tested welfare benefit available to all UK children and with near universal coverage. Participants were drawn from 398 randomly selected electoral wards in the United Kingdom. Sampling was geographically clustered and disproportionately stratified using the child poverty index to ensure that children in all four countries (England, Wales, Scotland and Northern Ireland) from disadvantaged and ethnic minority backgrounds were adequately represented (Plewis, 2007). To account for these aspects of methodology, weights for design, sampling and attrition were applied to all our analyses following the creation of a Complex Samples Plan. More details about the sampling strategy and the Complex Samples Procedure can be found elsewhere (Jones and Ketende, 2010; Hansen, 2012).

### Participants

The analysis includes respondents in MCS3 who were fathers of the cohort child at age 5 (*N* = 10 443) (including biological, adoptive, step and foster fathers). We decided to focus on this wave as differences in the development of children with and without ID may have become apparent by age 5, which could have a measurable impact on paternal well‐being. A targeted exploration of variables associated with paternal well‐being at one particular age range also allowed us to control for any developmental effects (Totsika, Hastings, Emerson, Berridge, and Lancaster, 2011).

Fathers may have been interviewed either as main or partner/second‐parental carer respondents, but in the majority of cases (96.2%), the father was the partner respondent. There were statistically significant differences between fathers with and without a child with ID on a range of demographic variables (Table 1). Fathers without a child with ID were more likely to be biological fathers, married to the other parent or carer in the household and described their ethnicity as White. Fathers also differed on a range of socio‐economic variables: fathers of children without ID were more likely to be in employment at the time of the research and have an educational qualification above degree level. They were also more likely to be full‐time residents in their child's home and not live in income poverty (below the 60% median equivilised income levels for the United Kingdom).

**Table 1:** Father demographics

Variables	Fathers of children without ID	Fathers of children with ID	
	Mean (SE)	Mean (SE)	*t*
Father age	37.45 (0.11)	36.80 (0.51)	1.58
Number of children in the home	2.39 (0.14)	2.90 (0.97)	−7.82*
Variables	%	%	*χ* ^2^
Father type			
Biological father	95.6	92.6	71.91*
Adoptive father	0.2	−	
Stepfather	4.2	7.4	
Foster father	−	−	
Relationship between parent/carers in the household			
Married	77.9	63.1	29.57*
Cohabiting	21.3	35.2	
Not applicable (including single, separated, divorced and widowed)	0.8	1.7	
Ethnic group			
White	90.0	82.1	82.03*
Mixed	0.7	–	
Indian	2.3	0.9	
Pakistani and Bangladeshi	3.7	14.9	
Black or Black British	1.7	2.1	
Other	1.6	–	
Employment status			
In work	91.4	78.4	47.77*
Not in work	8.6	21.6	
Educational level			
Degree level or above	41.5	18.8	44.41*
Below degree level	58.5	81.2	
Father resident in the child's home			
Full time	99.1	97.9	3.88*
Part time	0.9	2.1	
OECD poverty median indicator			
Above 60% median UK income	81.3	53.4	114.09*
Below 60% median UK income	18.7	46.6	

ID, intellectual disability; SD, standard deviation; SE, standard error

* *P* < 0.05.

All of the children in the analysis were 5 years of age; 5324 (51%) of the children were male (non‐ID = 5169, ID = 155) and 5119 (49%) female (non‐ID = 5018, ID = 101). Children with ID were reported to have higher levels of behavioural and emotional problems (M = 12.41, SE = 0.60) than children without ID (M = 6.59, SE = 0.07), and this difference was statistically significant (*t*(1) = −9.67, *P* < 0.001). To determine the presence of an ID in the cohort children, we adopted a grouping variable created in a study by Totsika *et al*. (in press) where ID was anchored at age 7 of the MCS (MCS4). A principal components analysis was conducted on age‐standardised scores on two subscales of the British Ability Scales Second Edition (Elliott *et al.,* 1996): pattern construction and word reading, and a mathematics test (NFER Progress in Maths). This confirmed the presence of an underlying factor representing the child's general cognitive ability (named ‘*g*') that accounted for 63% of the total variance. ID was defined as a *g* score equal or lower than two standard deviations below the mean (≤70). Where children could not be identified as having ID at age 7 due to missing data, a principal components analysis was conducted based on similar cognitive assessment data provided at age 5 (MCS3), age 3 (MCS2) or parent and teacher reported information at age 7 about ID if cognitive assessment data were unavailable at these time points. This method resulted in identifying 2.7% of MCS children with ID (weighted to account for the sampling design of MCS), which is consistent with the upper bound of estimates from a meta‐analysis of epidemiological research on children with ID (Maulik *et al.,* 2011).

### Measures

Paternal life satisfaction was measured using a single item that asked fathers to rate ‘how satisfied they were with the way their life had turned out so far' on a scale of 0 (*extremely unsatisfied*) to 10 (*completely satisfied*). This life satisfaction item is a subjective well‐being measure that has been used in national well‐being surveys by the UK Office for National Statistics. Scores can be categorised as very low (0–4), medium (5–6), high (7–8) and very high (9–10) (Office for National Statistics, 2017). Paternal work–family balance was measured using a single‐item measure that asked fathers to rate ‘their satisfaction with work/family balance' on a scale of 1 (*very satisfied*) to 5 (*very dissatisfied*). Fathers' general health was measured using a single‐item measure that asked fathers to rate their overall health on a 5‐point scale from 0 (*poor*) to 5 (*excellent*). The single‐item self‐rated health measure is one of the most commonly used measures of global health status (Krause and Jay, 1994) and has demonstrated reliability in clinical and research contexts (Bombak, 2013). Parenting self‐efficacy was measured using a single‐item measure that asked fathers to rate how they feel about being a parent on a 5‐point scale from 1 (*not very good at being a parent*) to 5 (*a very good parent*). Parent–child closeness was measured using a single‐item measure that asked fathers to rate their relationship with their child on a 4‐point scale from 1 (*not very close*) to 4 (*extremely close*). General health, parenting self‐efficacy and parent–child closeness all had negatively skewed distributions and so were dichotomised accordingly. General health scores were dichotomised into two groups: 0 = poor health (scores of 2 or lower) and 1 = good health (scores of 3 or higher). Parenting self‐efficacy scores were dichotomised into 0 = low level of parenting self‐efficacy (scores of 1 and 2) and 1 = high level of parenting self‐efficacy (scores of 3 or higher). Parent–child closeness was dichotomised into 0 = not close (scores of 1 and 2) and 1 = close (scores of 3 and 4).

Individual indicators of SEP (paternal education, paternal employment status and family income poverty) were dichotomised into degree/no degree education, in employment/not in employment, and families with an income above or below 60% of UK median equivalised income (as measured by the OECD). Individual predictors of SEP were favoured over a composite as we were particularly interested in the relationships between each predictor and the outcomes. A father residence variable was also included that indicated whether fathers were living in the same household as the child full time or part of the time.

The Strengths and Difficulties Questionnaire (SDQ; Goodman, 1997) was completed by primary caregivers for all the target cohort children as a measure of behavioural and emotional problems. Primary caregivers were mostly mothers; however, the current study also included SDQs completed by grandmothers. The 25‐item scale generates scores in four problem domains: emotional symptoms, conduct problems, hyperactivity and peer problems; with an additional pro‐social behaviour domain. Caregivers indicate how likely each statement (e.g. ‘Often unhappy, downhearted or tearful') applies to the child on a 3‐point scale: *Not true*, *somewhat true* and *very true*, based on their child's behaviour over the past 6 months. The SDQ is a reliable measure of behavioural and emotional problems and has been used in ID research (Totsika, Hastings, Emerson, Berridge, and Lancaster, 2011) and research with children without ID (Goodman, 2001). The SDQ total difficulties score was used in the present study (the sum of the four problem domains). A higher score indicates greater levels of behaviour and emotional problems. Internal consistency for this scale in our study was very good (Cronbach's α: children with ID: 0.97 and children without ID: 0.93).

### Procedure and analysis approach

Data for MCS3 were obtained from the UK Data Archive (www.ukdataservice.ac.uk). Ethical approval and informed consent had previously been obtained by the MCS research team. The initial step was to identify the total number of fathers in the MCS3 dataset, including fathers who were biological, adoptive, step and foster fathers, among those who had responded as a main or a partner respondent. The next stage was to identify how many of these fathers had a child with ID by combining father data with the child ID variable using the unique participant identifier. Data were available for 10 187 fathers of 5‐year‐old children without ID and 256 fathers of 5‐year‐old children with ID. Analyses were conducted in SPSS Statistics 24.0® using the Complex Samples Procedure.

To assess for differences between fathers of children with and without ID on aspects of psychological well‐being, we conducted independent *t*‐tests for life satisfaction and work–life balance and chi square tests for general health, parenting self‐efficacy and parent–child closeness measures. Two general linear regression models for life satisfaction and work–life balance, and three logistic regressions models for general health, parenting self‐efficacy and parent–child closeness measures, were then conducted to explore predictors of paternal well‐being.

## Results

### Comparing fathers' well‐being

Fathers of children with ID reported lower levels of life satisfaction compared with fathers of children without ID and the difference was statistically significant (*t*(1) = 2.46, *P* = 0.014), albeit small in terms of effect size (*d* = 0.17) (Table 2). There were no statistically significant differences between fathers of children with ID and fathers of children without ID on work–family balance (Table 2).

**Table 2:** t‐Test results for life satisfaction and work–family balance

	Fathers of children without ID		Fathers of children with ID			
	M	SE	M	SE	*t*	Cohen's *d*
Life satisfaction	7.59	0.02	7.10	0.20	2.46*	0.17
Work–family balance	2.94	0.01	3.00	0.12	−0.44	0.03

ID, intellectual disability

* *P* < 0.05.

There was a statistically significant difference in general health, with fathers of children without ID more likely to report good health compared with fathers of children with ID [*χ* ^2^ (1, *N =* 10 431) = 15.22, *P* < 0.001; Table 3]. There were no statistically significant differences between the two groups of fathers on the parenting self‐efficacy and parent–child closeness measures (Table 3).

**Table 3:** Chi square tests for general health, parenting self‐efficacy and parent–child closeness measures

		Fathers of children without ID		Fathers of children with ID		Adjusted *F* †
		%	Adjusted residual	%	Adjusted residual	
General health	Good	88.9	3.08	79.6	−3.08	15.22***
	Poor	11.1	−3.08	20.4	3.08	
Parenting self‐efficacy	High	96.0	0.75	94.9	−0.75	0.70
	Low	4.0	−0.75	5.1	0.75	
Parent–child closeness	High	90.1	0.85	87.9	−0.85	0.85
	Low	9.9	−0.85	12.1	0.85	

ID, intellectual disability

† Adjusted *F* (for categorical variables) = *F* statistic for design‐based Pearson chi square that is converted to *F* test to account for the MCS sampling design.

*** *P* < 0.001.

Scores for both groups of fathers were categorised as ‘high' for life satisfaction and ‘fairly satisfied' for work–family balance. The majority of fathers reported that their general health was ‘good' and that the relationship that they have with their child was ‘very close'. The majority of fathers of children with ID rated they felt that they were an ‘average parent', compared with the majority of fathers without ID who reported that they were a ‘better than average parent' on the parenting self‐efficacy measure (Table 4).

**Table 4:** Descriptive statistics on all dependent variables

	Fathers of children without ID			Fathers of children with ID		
	Mean (SE)	Range	Category	Mean (SE)	Range	Category
Life satisfaction	7.59 (0.02)	1–10	High	7.10 (0.19)	1–10	High
Work–family balance	2.94 (0.01)	1–5	Fairly satisfied	3.00 (0.12)	1–5	Fairly Satisfied
General health	3.71 (0.01)	1–5	Good	3.37 (0.07)	1–5	Good
Parenting self‐efficacy	4.00 (0.01)	1–5	>Average	3.92 (0.07)	1–5	Average
Father–child relationship	3.38 (0.00)	1–4	Very close	3.27 (0.05)	1–4	Very close

ID, intellectual disability; SE, standard error

### Factors associated with father well‐being

Regression models were conducted separately for each paternal outcome to explore the main effects of the following variables: whether the father had a child with ID, child gender, cohort child SDQ total difficulties score, father age, father education, father employment, father residency, family income poverty and number of the children in the household. Linear regression models were fitted for life satisfaction and work–life balance. Logistic regression models were fitted for general health, parenting self‐efficacy and parent–child closeness. As it is not possible to compute a single *R* ^2^ statistic using Complex Samples Procedure in SPSS, the Cox and Snell pseudo *R* ^2^ was used instead.

As shown by the unstandardised coefficients in Table 5, being in employment and living in a household not in income poverty were positively associated with life satisfaction. Child SDQ scores were negatively associated with life satisfaction: as child behavioural and emotional problems increased, life satisfaction scores decreased. Living in a household with a higher number of children was positively associated with life satisfaction scores. Child ID, child gender, father age, father education, and father residency were not statistically significant predictors. The overall model fit was pseudo *R* ^2^ = 0.022, a low level of explained variance in paternal life satisfaction scores.

**Table 5:** Linear regression models for life satisfaction and work–family balance

	Life satisfaction		Work–family balance	
	Coeff. (SE)	95% CI	Coeff. (SE)	95% CI
Constant	6.90*** (0.30)	[6.29, 7.49]	3.12*** (0.18)	[2.76, 3.48]
Child with ID	0.17 (0.22)	[−0.27, 0.62]	−0.16 (0.14)	[−0.44, 0.10]
Child gender	0.00 (0.04)	[−0.07, 0.09]	0.00 (0.02)	[0.05, 0.06]
Child SDQ	−0.02*** (0.00)	[−0.03, −0.01]	0.00* (0.00)	[0.00, 0.01]
Father age	−0.00 (0.00)	[−0.01, 0.00]	−0.00** (0.00)	[−0.01, −0.00]
Father education	−0.04 (0.04)	[−0.13, 0.04]	0.01 (0.03)	[0.04, 0.08]
Father employment	0.53*** (0.11)	[0.29, 0.76]	–	–
Father residency	−0.40 (0.25)	[−0.90, 0.09]	−0.10 (0.17)	[−0.45, 0.22]
Income poverty	0.36*** (0.07)	[0.22, 0.51]	0.18 (0.05)***	[0.08, 0.28]
Number of children in household	0.06** (0.00)	[0.01, 0.11]	0.01 (0.01)	[−0.1, 0.05]

ID, intellectual disability; SDQ, Strengths and Difficulties Questionnaire; SE, standard error

* *P* < 0.05.

** *P* < 0.01.

*** *P* < 0.001.

Living in a household not in income poverty and child SDQ scores were positively associated with work–family balance (Table 5). Father age was negatively associated with work–family balance. Child ID, child gender, father education, father residency, and the number of children in the household were not associated with work–family balance (Table 5). Father employment was not included in this model because the work–family balance measure was completed by working fathers only. The overall model fit was pseudo *R* ^2^ = 0.005, a low level of explained variance.

Child SDQ scores and father age were significantly associated with general health (Table 6). Fathers of children with higher behavioural and emotional problems and older fathers were more likely to report poor levels of general health. A father's education status, employment status and family income poverty level were also significantly associated with general health. Fathers who possessed a degree level qualification were more likely to report poor levels of general health. Fathers in work and not living in income poverty were less likely to report poor general health. Child ID, child gender, father residency, and number of children in the household were not statistically significant predictors. The overall model fit was pseudo *R* ^2^ = 0.047, a low level of explained variance.

**Table 6:** Logistic regression model for general health

	*B*	SE	Wald	*P*	OR	OR 95% CI
Constant	−2.51	0.40	87.57	<0.001		
Child with ID	−0.03	0.27	0.01	0.897	0.97	[0.57, 1.66]
Child gender	0.00	0.09	0.01	0.922	1.00	[0.84, 1.21]
Child SDQ score	0.04	0.00	29.18	<0.001	1.04	[1.02, 1.06]
Father age	0.02	0.00	11.37	0.001	1.02	[1.01, 1.04]
Father education	0.56	0.10	27.78	<0.001	1.75	[1.41, 2.15]
Father employment	−1.25	0.11	113.05	<0.001	0.28	[0.22, 0.36]
Father residency	−0.39	0.42	0.88	0.350	0.68	[0.29, 1.55]
Income poverty	−0.41	0.11	13.65	<0.001	0.67	[0.54, 0.83]
Number of children in household	0.06	0.04	2.41	0.121	1.07	[0.99, 1.16]

*B*, estimated value of the regression coefficient; *df*, degrees of freedom; ID, intellectual disability; OR, odds ratio; *P*, level of significance; SDQ, Strengths and Difficulties Questionnaire; SE, standard error; Wald, Wald statistic; 95% CI, 95% confidence interval

Child SDQ scores, father age and father employment were significantly associated with parenting self‐efficacy (Table 7). Fathers of children with higher behavioural and emotional problems and older fathers were more likely to report low‐parenting self‐efficacy. Fathers in work were less likely to report low‐parenting self‐efficacy. Child ID, child gender, father education, father residency, income poverty and number of children in the household were not statistically significant predictors (Table 7). The overall model fit was pseudo *R* ^2^ = 0.005, a low level of explained variance.

**Table 7:** Logistic regression model for parenting self‐efficacy and parent–child closeness

	Parenting self‐efficacy						Parent–child closeness					
	*B*	SE	Wald	*P*	OR	OR 95% CI	*B*	SE	Wald	*P*	OR	OR 95% CI
Constant	−0.432	0.62	73.38	<0.001			−3.53	−0.48	62.00	<0.001		
Child with ID	0.26	0.36	0.55	0.461	1.30	[0.65, 2.65]	0.34	0.27	1.57	0.211	1.41	[0.82, 2.45]
Child gender	0.10	0.12	2.14	0.144	1.19	[0.95, 1.53]	0.03	0.08	0.22	0.636	1.03	[0.89, 1.21]
Child SDQ score	0.04	0.01	10.85	0.001	1.04	[1.01, 1.06]	0.06	0.00	80.81	<0.001	1.06	[1.05, 1.08]
Father age	0.02	0.00	8.17	0.004	1.02	[1.00, 1.04]	−0.00	0.00	0.01	0.921	1.00	[0.99, 1.01]
Father education	−0.03	0.12	0.08	0.778	0.97	[0.76, 1.23]	−0.05	0.08	0.47	0.490	0.95	[0.80, 1.11]
Father employment	−0.54	0.27	3.09	0.049	0.59	[0.34, 1.00]	0.37	0.19	3.85	0.051	1.45	[1.00, 2.10]
Father residency	0.60	0.49	1.45	0.228	1.83	[0.69, 4.83]	0.35	0.34	1.05	0.305	1.42	[0.73, 2.82]
Income poverty	−0.10	0.18	0.32	0.572	0.91	[0.63, 1.29]	−0.29	0.11	6.82	0.009	0.75	[0.60, 0.93]
Number of children in household	0.05	0.06	0.73	0.395	1.06	[0.93, 1.21]	0.17	0.04	16.40	<0.001	1.19	[1.09, 1.30]

*B*, estimated value of the regression coefficient; *df*, degrees of freedom; ID, intellectual disability; OR, odds ratio; *P*, level of significance; SDQ, Strengths and Difficulties Questionnaire; SE, standard error; Wald, Wald statistic; 95% CI, 95% confidence interval

In the parent–child closeness model (Table 7), number of children in the household, child SDQ scores and income poverty were statistically significant predictors. Fathers with more children in the household and fathers of children with higher behavioural and emotional problems were more likely to report a poor relationship with their child. Fathers who did not live in income poverty were less likely to report a poor relationship with their child. Child ID, child gender, father age, father education, father employment and father residency were not statistically significant predictors (Table 7). The overall model fit was pseudo *R* ^2^ = 0.015, a low level of explained variance.

## Discussion

The study is among the first to compare fathers of children with and without ID in a population‐based sample on a range of well‐being and parenting measures. It is important to compare the well‐being of fathers of children with and without ID using UK representative data to further understand the impact of raising a child with ID on paternal well‐being and develop interventions that serve a larger population of fathers of children with ID and their families.

Our first aim was to explore whether fathers of children with and without ID differed on well‐being and parenting measures. Comparative analyses showed that there were small differences between fathers, with fathers of children with ID reporting lower life satisfaction and poorer general health outcomes. Previous evidence has indicated that fathers of children with disabilities report lower life satisfaction (Darling *et al.,* 2012) and poorer general health outcomes (Seymour *et al.,* 2017) compared with fathers of children without disabilities. However, it is important to note that these studies are not directly comparable as they did not focus specifically on fathers of children with ID.

The finding that fathers of children with ID are reporting poorer on well‐being measures beyond psychological distress extends our knowledge. Reports of lower life satisfaction and poorer general health outcomes demonstrate that there is a need to also explore the impact of raising a child with ID on these broader aspects of well‐being and indeed consider what can be done to support fathers in these domains. As the developmental systems model suggests (Guralnick, 2005), parental outcomes can have an impact on the outcomes of children; thus, future research might fruitfully begin to focus on the associations between these paternal well‐being variables and the developmental outcomes of children with ID. However, it is also important to consider the variables where there were no significant differences. While we might have expected differences in work–family balance, parenting self‐efficacy and father–child closeness (related to increased caregiving demands), our findings indicate that, when the child is 5 years of age, having a child with ID is not associated with poorer outcomes in these areas. Further exploration of the descriptive statistics showed that overall fathers of children with ID had similar scores to fathers of children without ID on almost all of the measures. This rejects the prevailing simple narrative that raising a child with a disability negatively and uniformly affects parental well‐being (Hastings, 2016; Totsika *et al.,* 2016; Seymour *et al.,* 2017; Weiss *et al.,* 2018).

The second aim of the study was to explore whether the presence of a child with ID in the family was a significant predictor of paternal well‐being when controlling for a number of other variables. Our findings showed that having a child with ID was not a significant predictor of any of the paternal well‐being variables, indicating that the differences between the two groups of fathers in the initial analysis may not be associated directly with having a child with ID. Similar findings have also been reported with fathers of children with autism, where child characteristics were not significantly associated with variance in fathers' psychological distress (Seymour *et al.,* 2018). However, our findings are in contrast to previous findings from other population‐representative studies with mothers with ID (Totsika, Hastings, Emerson, Lancaster, and Berridge, 2011) and early cognitive delay (Emerson *et al.,* 2010), indicating that well‐being outcomes could, in theory, be differentially determined for mothers and fathers of children with ID. This may be related to role specialisation where mothers are more likely to be engaged in childcare (Hartley *et al.,* 2014; Eagly and Wood, 2016), or there are other factors such as income and employment that are stronger predictors of well‐being in fathers, which has also been found in research with fathers in the general population (Cooklin *et al.,* 2015).

Child behavioural and emotional problems were found to be predictive of all of the well‐being measures in this study, supporting other studies that have explored the association between child behaviour and paternal psychological distress (Herring *et al*. 2006), depression, anxiety (Giallo *et al.,* 2015; Cohen *et al.,* 2016) and stress (Ricci and Hodapp, 2003; Brobst *et al.,* 2009). Notably, child behaviour problems were also negatively associated with the two parenting outcomes: parenting self‐efficacy and the father–child relationship, corroborating previous work that has indicated an association between behaviour problems and parenting self‐efficacy in parents of children with ID (Hassall *et al.,* 2005). This is something that could be ameliorated with the right support; family‐focused interventions could be examined that seek to develop the skills and knowledge of fathers to support their child's behavioural and emotional problems, facilitate positive father–child interactions and provide space for fathers to reflect on how their child's behaviour affects their well‐being, parenting and relationships.

Like previous studies (Emerson and Llewellyn, 2008; Emerson *et al.,* 2010), there was an association between socio‐economic factors and paternal well‐being. Living in income poverty was a strong predictor of a number of paternal well‐being outcomes, with fathers who reported living in income poverty reporting lower life satisfaction, work–life balance and general health. Living in income poverty limits choice and opportunity and is likely to place greater pressure on a father to provide for their family and dictate the amount and type of work they do (Wright *et al.,* 2016). Living in income poverty was also found to be associated with the closeness of the father–child relationship. Stresses and strains associated with living in poverty are likely to infiltrate family relations (Totsika *et al.,* in press), as well as have the potential to affect the ability of the father to do things with their child that foster a positive relationship. Fathers of children with ID may be more vulnerable to financial hardship and are more likely to experience greater economic strain due to reduced family earning capacity and the additional costs associated with raising a child with a disability (Stabile and Allin, 2012). There is also evidence to suggest that families of a disabled child are more likely to be in persistent or recurrent poverty (Shahtahmasebi *et al.,* 2011). These findings can be understood within the context of family systems theories. ‘Variety', the extent to which a ‘system has the resources to meet new environmental demands' (White *et al*., 2015, p.150), is proposed as necessary for families to adapt to challenges and ensure system equilibrium. However, if families do not have much variety (e.g. the financial support needed to support their child with ID and their family), then these findings suggest that this could have implications for paternal well‐being and parenting outcomes. Providing greater financial support to families, in particular to help with expenses related to raising a child with ID, would be a positive step in improving the lives of fathers and likely, in turn, their children.

Employment was also associated with paternal well‐being. Fathers in employment had higher levels of life satisfaction and were less likely to report poor general health, echoing findings in the general population where experiencing unemployment has been found to be negatively associated with well‐being outcomes, markedly reducing life satisfaction (Pittau *et al.,* 2010). Fathers in work were also less likely to report low‐parenting self‐efficacy. Work provides individuals with the chance to set goals and achieve them, increasing self‐esteem and a sense of competence (Erdogan *et al.,* 2012), which could spill over into parenting. Further work is needed to explore the associations between employment and paternal well‐being. However, it may be also useful to explore job satisfaction in addition to whether a father is in employment.

The current study does have some limitations. First, paternal outcomes were measured by self‐report; thus, future research might consider observational studies to reduce the likelihood of socially desirable responses, especially for parenting and relationship outcomes, although this would be harder to achieve on a large scale. The behaviour of the child was also predominantly reported by mothers. This is a strength of the study design because it provides some independence of measurement. Where the measure of the predictor and criterion variable has been provided by the same respondent, this has been found to complicate the apportioning of variance to independent factors (Podsakoff *et al.,* 2003). Watson and Clark (1984) describe the issue of ‘mood state' where respondents who view themselves as generally positive or negative view the world around them in the same way. However, we do acknowledge that maternal reports may not provide the best measure of fathers' exposure to their child's behavioural and emotional problems. Single‐item measures may also be less reliable in capturing the variable of interest (Seymour *et al.,* 2017); however, they have been used in large‐scale studies such as the MCS as they are less burdensome for participants (Zimmerman *et al.,* 2006) and are often very effective. Skewed data were an issue in the current analysis, so the use of multi‐item measures may be a way to address this in the future. It may also be that for some aspects, such as work–family balance, we need to investigate more specifically the conditions of paternal employment (i.e. satisfaction and flexibility) that are more likely to be affected by raising a child with a disability. Lastly, it is also important to note that the present analysis focused on fathers living in the same household as the child for all or part of the time, and, therefore, findings cannot be generalised to fathers who were not living in the same household as the child at the time of data collection.

While cross‐sectional analyses have allowed us to understand some of the factors associated with paternal well‐being at this particular stage of their child's life cycle (age 5), longitudinal research is needed to understand whether a similar pattern of findings is seen at different ages and the trajectories of well‐being in these group of fathers. There is also a need to explore father‐to‐child effects, exploring the impact of paternal well‐being and parenting on the development of children with ID. More broadly, fathers are still heavily underrepresented in family research (Phares *et al.,* 2005; Cassano *et al.,* 2006), resulting in significant gaps in knowledge and clinical application (Seymour *et al.,* 2018). Further exploratory work is required as to why fathers may not participate in research as readily as mothers.

Moving forward, our findings, which show that only a minority of fathers with ID seem to be at increased risk for poorer well‐being, indicate that universal intervention is not warranted. However, there is a need to focus on fathers where their child has significant levels of behavioural and emotional problems, and they are living in income poverty. Fathers experiencing these risk factors might require targeted psychological support and help from services to improve their capacity to care for, and engage effectively with, their child.

## Source of funding

This work was funded by an Economic and Social Research Council scholarship (1501957). This research has also been part funded by the charity Cerebra.

## Conflict of interest

No conflicts of interest have been declared.
